# Transmission dynamics of lyssavirus in *Myotis myotis*: mechanistic modelling study based on longitudinal seroprevalence data

**DOI:** 10.1098/rspb.2023.0183

**Published:** 2023-04-26

**Authors:** Younjung Kim, Stefania Leopardi, Dino Scaravelli, Barbara Zecchin, Pamela Priori, Francesca Festa, Petra Drzewnioková, Paola De Benedictis, Pierre Nouvellet

**Affiliations:** ^1^ Department of Evolution, Behaviour, and Environment, School of Life Sciences, University of Sussex, BN1 9RH Brighton, UK; ^2^ FAO and National Reference Centre for Rabies, Istituto Zooprofilattico Sperimentale delle Venezie, Viale dell'Università 10, Legnaro, 35020 Padua, Italy; ^3^ S.T.E.R.N.A. and Museo Ornitologico ‘F. Foschi’, via Pedrali 12, 47121 Forlì, Italy; ^4^ Department of Biological, Geological and Environmental Sciences, University of Bologna, via Selmi 3, 40126 Bologna, Italy; ^5^ Department of Infectious Disease Epidemiology, School of Public Health, Imperial College London, SW7 2AZ London, UK

**Keywords:** lyssavirus, EBLV-1, *Myotis* bats, bat-borne virus, zoonosis, transmission dynamics

## Abstract

We investigated the transmission dynamics of lyssavirus in *Myotis myotis* and *Myotis blythii*, using serological, virological, demographic and ecological data collected between 2015 and 2022 from two maternity colonies in northern Italian churches. Despite no lyssavirus detection in 556 bats sampled over 11 events by reverse transcription-polymerase chain reaction (RT-PCR), 36.3% of 837 bats sampled over 27 events showed neutralizing antibodies to *European bat lyssavirus 1*, with a significant increase in summers. By fitting sets of mechanistic models to seroprevalence data, we investigated factors that influenced lyssavirus transmission within and between years. Five models were selected as a group of final models: in one model, a proportion of exposed bats (median model estimate: 5.8%) became infectious and died while the other exposed bats recovered with immunity without becoming infectious; in the other four models, all exposed bats became infectious and recovered with immunity. The final models supported that the two colonies experienced seasonal outbreaks driven by: (i) immunity loss particularly during hibernation, (ii) density-dependent transmission, and (iii) a high transmission rate after synchronous birthing. These findings highlight the importance of understanding ecological factors, including colony size and synchronous birthing timing, and potential infection heterogeneities to enable more robust assessments of lyssavirus spillover risk.

## 1. Introduction

In recent years, bats have received considerable attention as a reservoir of zoonotic viruses [1–7]. One notable feature frequently observed in bat-borne viruses is distinct seasonal fluctuations in their prevalence or seroprevalence [1,8–10]. However, the mechanisms responsible for such seasonal patterns remain poorly understood, making it challenging to reliably assess lyssavirus transmission dynamics, identify risk factors and ultimately estimate zoonotic spillover risk [11].

Mechanistic modelling is particularly well suited to understanding the mechanisms underpinning temporal infectious disease transmission trends by explicitly describing biological and ecological processes hypothesized to drive transmission dynamics and then testing their significance based on longitudinal surveillance data. However, most studies interested in the seasonality of viral transmission among bats remained descriptive [1,8–10,12,13], and only a few studies investigated the mechanisms driving the seasonality by fitting mechanistic models to virological or serological data [14–18], possibly owing to challenges in conducting longitudinal surveys in bat populations with sufficient temporal resolution and sample size. This is also true for research into lyssaviruses, which are responsible for a notable zoonotic disease, rabies.

Although the global burden of rabies is mainly caused by dog-associated rabies virus (RABV), other lyssaviruses are also capable of causing encephalitis in humans and animals typically observed with RABV infections [19]. Bats have special significance among reservoir hosts in lyssavirus epidemiology and have been found to harbour RABV in the Americas and other lyssaviruses across Africa, Eurasia and Australia. Furthermore, bats serve as the ancestral hosts of most lyssaviruses currently circulating in other animal species [20]. Consequently, bat rabies is an emerging public and animal health concern in the Americas, accounting for most of the human rabies cases there [19], and worldwide including Europe, where sporadic infections in humans and other animals have been reported [21–29].

In European bats, six lyssaviruses have been so far identified, namely, European bat lyssavirus 1 (EBLV-1) and 2 (EBLV-2), Bokeloh bat lyssavirus, West Caucasian bat virus, Lleida bat lyssavirus and Kotalahti bat lyssavirus [20]. Of these viruses, EBLV-1 is most frequently reported in continental Europe [20] and has recently been reported in Great Britain [30], with sporadic spillover events to humans and non-flying mammals [22–24,31]. Serotine bats, including *Eptesicus serotinus* and *Eptesicus isabellinus*, have been long considered the reservoir of EBLV-1. However, virological and serological evidence has also been found in several other bat species, including greater mouse-eared bats, i.e. *Myotis myotis* (Borkhausen, 1797), in different countries in Europe [32–36].

Although lyssaviruses have been suggested to circulate among European bats, their transmission dynamics are poorly understood. Three studies investigated factors contributing to long-term viral persistence in bat populations by simulating disease spread over a range of mechanistic models and parameter values collated from the literature [37–39]. However, while providing valuable theoretical insights based on simulation, these studies did not explicitly test hypotheses on lyssavirus transmission mechanisms by fitting their models to disease surveillance data.

Here, we aimed to understand drivers for lyssavirus transmission in two mouse-eared species based on longitudinal surveillance data generated between 2015 and 2022 from two maternity colonies in northern Italian churches comprising *M. myotis* and *Myotis blythii* (Tomes, 1857). First, we examined risk factors for lyssavirus seropositivity and characterized its seasonal trends within and between years. Then, we investigated how key elements in infectious disease epidemiology, associated with the bats' life cycle, drive lyssavirus transmission by fitting comprehensive sets of mechanistic models to lyssavirus seroprevalence data.

## 2. Methods

### (a) Data

The transmission dynamics of lyssavirus were investigated based on longitudinal lyssavirus surveillance data generated from two roosts located in northeast Italy between 2015 and 2022. These roosts, referred to as colony 1 and colony 2, were located under the roof of churches and inhabited by a mixed population of two sister species, i.e. *M. myotis* and *M. blythii*, from April to October each year. Both colonies were classified as maternity colonies, consisting mostly of adult females that gave birth almost synchronously in late May. Adult males were only occasionally observed, mainly during the mating season in late summer.

Bat sampling was approved by the Italian Ministry for the Environment in derogation to the Council Directive 92/43/EEC of 21 May 1992 on the conservation of natural habitats and of wild fauna and flora (authorization nos. 9749-12, 7588-18, 23986-21, 13848-22). In both colonies, bats roosted on the timbers or stone walls inside the church roof (electronic supplementary material, figure S1). Multiple photos were taken at each sampling event to capture the areas where bats roosted with a sufficient resolution for counting bats. Colony size was estimated by summing the number of bats counted from these photos (electronic supplementary material, figure S1). Bats were captured from the roof during the day using hand nets and placed in cotton bags for 5–10 min before sampling. For each captured bat, 50–200 µl of blood was collected from the interfemoral vein, and for a subset of captured bats, salivary swabs and biopsies from the interfemoral patagium were collected. Blood samples were tested by the rapid fluorescent focus inhibition test to detect neutralizing antibodies against lyssaviruses, using EBLV-1 as the challenge virus. Salivary swab samples were tested for lyssaviruses by reverse transcription-polymerase chain reaction (RT-PCR), following previously described protocols [32,40]. Species, age, sex and physiological status were determined for each captured bat. *Myotis myotis* and *M. blythii* were primarily distinguished morphologically by the length of the upper tooth row between canines and molars [41], and genetic confirmation was performed for 141 bats by sequencing the mitochondrial cytochrome *b* gene, using the biopsies as matrices for DNA extraction [42]. Bats were classified as adult (i.e. one year or older) if no visible remaining cartilage was present between the epiphyseal plates of their wing long bones [43]. Otherwise, they were classified as young (i.e. less than one-year old).

The longitudinal surveillance data generated between 2015 and 2021 through the above processes were used for three purposes. First, we investigated risk factors for lyssavirus seropositivity using mixed-effect logistic regression models. Then, we characterized the seasonal variability in lyssavirus seroprevalence using a binomial generalized additive model (GAM), and finally, we examined mechanisms underlying lyssavirus transmission based on mechanistic compartmental models.

Separately in 2022, salivary swab samples were collected from 200 bats on the date suggested to be in the main transmission season by our modelling approaches described below and tested by RT-PCR for lyssavirus detection; 200 was the maximum number of bats that could be sampled in one day for ethical and logistical considerations.

### (b) Risk factors for lyssavirus seropositivity

We explored the association of lyssavirus seropositivity with demographic (i.e. *sex*, *age* and *species*) and physiological (i.e. *physiological status*) factors using multivariate mixed-effect logistic regression analysis. *Sampling year* and *colony* were included as random effects assuming correlation in lyssavirus transmission within sampling years and colonies. Each year, only adult bats were sampled before July when lyssavirus seroprevalence tended to be low, whereas from July, when lyssavirus seroprevalence was relatively high, both young and adult bats were sampled. Therefore, samples collected after 1 July were included in mixed-effect logistic regression analysis to avoid potential bias from temporal variability in lyssavirus transmission within sampling years. The mixed-effect logistic regression analysis was conducted using the *glmer* function of the *lmer4* package [44] in R. 4.1.2 [45].

### (c) Seasonal trends in lyssavirus seroprevalence

Seasonal trends in lyssavirus seroprevalence were analysed by a GAM, which included (i) a cubic spline term for intra-annual variability, (ii) a random effect term for inter-annual variability, and (iii) a random effect term for colony variability. The covariates *age*, *sex*, *species*, and *physiological status* were also added as fixed effect terms. GAM analysis was conducted using the *gam* function of the *mgcv* package [46] in R. 4.1.2 [45].

### (d) Lyssavirus transmission dynamics in *Myotis myotis* maternity colonies

#### (i) Baseline model

We fitted mechanistic compartmental models to longitudinal seroprevalence data collected from *M. myotis* bats from the two colonies between 2015 and 2021. We explored two sets of compartment models, which we refer to as SEIR and SEID/SER models. SEIR models assumed a homogeneous lyssavirus infection where all bats experience transitions from susceptible (S), exposed (E), infectious (I) and recovered with immunity (R) after viral exposure. By contrast, SEID/SER models assumed a heterogeneous lyssavirus infection where, upon exposure, a fraction of bats (E_1_) become infectious (I) and then die (D), while the remaining bats (E_2_) recover with immunity (R) without being infectious, as commonly assumed in bat-RABV studies [16,47,48]. In both model sets, each colony was structured into young and adult bats, and the following assumptions were made on *M. myotis* population dynamics based on field observations. First, we assumed that each year, adult bats formed maternity colonies on 1 April and gave synchronous birth on 25 May, and all bats hibernated from 14 October. Second, considering that *M. myotis* generally has one offspring each year [49], we assumed that, after synchronous birthing, adult bats comprised half of the total population, with young bats comprising the remaining half. Each year, the largest number of bats estimated across sampling events was assumed to be the colony population size. Finally, at the beginning of 2015, in both colonies, all bats were assumed susceptible (S), except for one exposed (E) and one infectious (I) adult bat. This was based on the low seroprevalence observed on 15 May 2015, in adult bats in colony 1 (i.e. mean: 1.6%, 95% confidence interval (CI): 0–8.5%). No data were available for 2015 in Villa, but we assumed that both colonies experienced similar epidemic trends as observed in other years. Accordingly, all young bats were assumed born as susceptible in 2015. Then, at the beginning of the maternity colony forming in the following years, the proportion of adult bats in each compartment followed the proportion at the end of hibernation in the preceding year. Likewise, the proportion of young bats born with maternal immunity followed the proportion of adults with immunity at the end of hibernation in the preceding year. Finally, maternal immunity was assumed to wane faster than immunity from infection [50].

The baseline SEIR model was constructed by equations (2.1)–(2.10):

2.1 $$\frac{dS_{c,a}(t)}{dt}=m_{S_{c}}+\sigma R_{c,a}(t)-\lambda_{c}(t)S_{c,a}(t)-\delta S_{c,a}(t),$$

2.2 $$\frac{dE_{c,a}(t)}{dt}=m_{E_{c}}+\lambda_{c}(t)S_{c,a}(t)-\delta E_{c,a}(t)-\tau E_{c,a}(t),$$

2.3 $$\frac{dI_{c,a}(t)}{dt}=m_{I_{c}}+\tau E_{c,a}(t)-\delta I_{c,a}(t)-\gamma I_{c,a}(t),$$

2.4 $$\frac{dR_{c,a}(t)}{dt}=m_{R_{c}}+\gamma I_{c,a}(t)-\delta R_{c,a}(t)-\sigma R_{c,a}(t),$$

2.5 $$\frac{dS_{c,y}(t)}{dt}=b_{S_{c}}+\sigma R_{c,y}(t)+(\sigma +\phi )M_{c}(t)-\lambda_{c}(t)S_{c,y}(t)-\delta S_{c,y}(t),$$

2.6 $$\frac{dE_{c,y}(t)}{dt}=\lambda_{c}(t)S_{c,y}(t)-\delta E_{c,y}(t)-\tau E_{c,y}(t),$$

2.7 $$\frac{dI_{c,y}(t)}{dt}=\tau E_{c,y}(t)-\delta I_{c,y}(t)-\gamma I_{c,y}(t),$$

2.8 $$\frac{dR_{c,y}(t)\;}{dt}=\gamma I_{c,y}(t)\;-\delta R_{c,y}(t)\;-\sigma R_{c,y}(t),$$

2.9 $$\mathrm{and}\;\frac{dM_{c,y}(t)\;}{dt}=b_{M_{c}}-\delta M_{c,y}(t)\;-(\sigma +\phi )M_{c}(t).$$

Model parameters and assumptions are summarized in table 1 and the electronic supplementary material, table S1, respectively. Subscript *c* denotes a maternity colony, and *y* and *a* denote young and adult bats, respectively. *m* represents adult bats arriving to form maternity colonies, and *b* represents the influx of young bats through a synchronous birth pulse. The subscripts of *m* and *b* denote the compartments where bats belong. *δ* is the rate of natural death provided as the inverse of the average lifespan of *M. myotis*. *τ* is the rate of becoming infectious; *γ* is the rate of recovering and becoming immune; *σ* is the rate of loss of immunity from infection; the loss of maternal immunity is modelled as *σ* + ϕ to allow maternal immunity to wane faster than immunity from infection.

**Table 1.** Model hypotheses and comparison results

model	baseline and alternative hypotheses	SEIR (final models in bold)			SEID/SER (final models in bold)		
		DIC	DICΔ^a^	model weight^b^	DIC	DICΔ^a^	model weight^b^
baseline model	• density-dependent transmission • transmission rate (*β*) ◦ no transmission during hibernation (*β* = 0) *β* changes in a stepwise manner at the start of the following life cycle events: 1) maternity colony forming, 2) synchronous birthing, 3) hibernation rate of becoming infectious (*τ*), rate of recovering and becoming immune (*γ* in SEIR, ϑ in SEID/SER) *τ*, *γ*, and ϑ are constant non-zero during hibernation ◦ *τ*, *γ*, and ϑ are zero during hibernation • rate of immunity loss (*σ*) changes in a stepwise manner at the start of hibernation. • no recrudescence	283.8	47.2	0.00	277.7	41.1	0.00
model 1	frequency-dependent transmission	318.9	82.3	0.00	324.9	88.3	0.00
model 2.1	*β* changes following a sinusoidal curve until hibernation.	**236.6**	**0**	**0.59**	**239.2**	**2.6**	**0.16**
model 2.2	*β* is constant until hibernation.	377.0	140.4	0.00	354.6	118	0.00
model 3.1	*σ* changes throughout each year following a sinusoidal curve	280.8	44.2	0.00	269.1	32.5	0.00
model 3.2	*σ* is constant throughout each year	335.2	98.6	0.00	299.9	63.3	0.00
model 4	no maternal immunity	291.9	55.3	0.00	314.3	77.7	0.00
model 5	*τ*, *γ*, and ϑ are constant non-zero throughout the year	**240.8**	**4.2**	**0.07**	243.4	6.8	0.02
model 6.1	• model 5 + *β* changes following a sinusoidal curve until hibernation, • no transmission during hibernation (*β* = 0)	**240.8**	**4.2**	**0.07**	257.4	20.8	0.00
model 6.2	model 5 + *β* changes following a sinusoidal curve throughout the year, including during hibernation (*β* ≠ 0)	261.8	25.2	0.00	264.0	27.4	0.00
model 6.3	model 5 + *β* during hibernation is estimated separately from other periods (*β* ≠ 0)	**240.8**	**4.2**	**0.07**	243.3	6.7	0.02
model 6.4	model 5 + *β* is constant throughout the year, including during hibernation (*β* ≠ 0)	415.0	178.4	0.00	355.8	119.2	0.00
model 7	bats with immunity become infectious again at the rate of *ρ*	283.9	47.3	0.00	NA		
model 8	death from infection at the rate of *χ*	283.9	47.3	0.00	NA		

^a^Difference from the lowest DIC (i.e. 236.6 from SEIR model 2.1).

^b^Model weights were obtained using exp(-|DICΔ|/2) and standardised over all models compared (sum to 1) [54].

In the baseline model, we assumed that young and adult bats lost immunity from natural infection at the same rate, *σ* (i.e. ϕ = 0). We assumed that exposed (E) or infectious (I) bats remained in the same compartment during hibernation (i.e. *τ*, ϑ, and *γ* = 0 during hibernation). In addition, *σ* was estimated separately before and during hibernation, considering potentially altered physiological status during hibernation [52,53].

The force of infection, *λ*, was modelled as density-dependent in the baseline model through the transmission rate, *β*:

2.10 $$\lambda_{c}(t)\;=\beta (I_{c,y}(t)\;+I_{c,a}(t)),$$

where *β* was modelled to change in a stepwise manner synchronized with the following life cycle events: (i) a return to a maternity colony, (ii) synchronous birth pulse, and (iii) hibernation start. Finally, no transmission was assumed during hibernation (i.e. *β* = 0)

The baseline SEID/SER model was constructed in the same way as the baseline SEIR model, except that, upon exposure, bats were allowed to become infectious and die with probability π (E_1_ to I to D) or recover with immunity without becoming infectious with probability 1−π (E_2_ to R). Here, the rate of becoming infectious (E_1_ to I) was assumed different from the rate of recovering with immunity without becoming infectious (E_2_ to R) (see the electronic supplementary material, table S1 and for model specifications).

#### (ii) Alternative models

Both baseline SEIR and SEID/SER models were compared with alternative models to test the following hypotheses on lyssavirus transmission mechanisms (table 1).

***Hypothesis 1*** *on the force of infection:* the transmission was assumed to be frequency-dependent by modelling *λ* through the transmission rate, *ε* (model 1):

2.11 $$\lambda_{c}(t)\;\;=\frac{\varepsilon (\;I_{c,y}(t)+I_{c,a}(t))}{N_{c}(t)}.$$

***Hypothesis 2*** *on the transmission rate, β:* first, *β* was assumed to change continuously during non-hibernation periods following a sinusoidal curve, reaching the highest and lowest levels at different times (model 2.1):

2.12 $$\beta (t)=\beta_{0}+\frac{\beta_{1}(\sin(2\pi (t-\alpha ))+1))}{2},$$

where *β*_0_ represents the minimum *β*, and *β*_1_ represents the amplitude of seasonality in *β* (i.e. maximum increase in *β*). *α* represents the timing of seasonality and was estimated along with *β*_0_ and *β*_1_. Like in the baseline model, no transmission was assumed during hibernation (i.e. both *β*_0_ and *β*_1_ = 0 during hibernation).

Second, we assumed that *β* was constant until hibernation (model 2.2).

***Hypothesis 3*** *on the rate of immunity loss:* first, the rate at which immunity from natural infection was lost, *σ*_,_ was assumed to change continuously throughout each year with distinct highest and lowest peaks (model 3.1):

2.13 $$\sigma (t)=\sigma_{0}+\frac{\sigma_{1}(\sin(2\pi (t-\omega ))+1))}{2},$$

where *σ*_0_, *σ*_1_ and ω represent the minimum *σ*, the amplitude of seasonality in *σ* (i.e. maximum increase in *σ*), and the timing of seasonality in *σ*, respectively. The additional rate for maternal immunity loss, *ϕ*, was modelled to be constant, assuming that the loss of maternal immunity follows the trend in the loss of immunity from infection. Alternatively, *σ* was assumed to be constant throughout the years, instead of being assumed to be different before and during hibernation (model 3.2).

***Hypothesis 4*** *on maternal immunity:* all young bats were assumed susceptible at birth (i.e. no maternal immunity, *b*_M_=0) (model 4).

***Hypothesis 5*** *on the rate of becoming infectious, τ*, *and the rate of recovering and becoming immune* *(γ* *in the SEIR model, ϑ in the SEID/SER model):* *τ*, *γ* and ϑ were assumed constant throughout the year, i.e. *τ*, *γ* and ϑ ≠ 0 (model 5).

***Hypothesis 6*** *building upon Hypothesis 5, on the transmission during hibernation:* in models 6.1-4, the infection status was also assumed to change during hibernation (*hypothesis 5*). First, *β* was modelled to follow a sinusoidal curve, as model 2.1, with *β*_0_ and *β*_1_ during hibernation fixed at zero (model 6.1) or not (model 6.2). When a stepwise change was assumed, *β* during hibernation was estimated as a separate parameter from *β* in other periods (model 6.3) or assumed constant throughout the year (model 6.4).

***Hypothesis 7*** *on recrudescence*: after recovery, bats were assumed to become infectious again at the rate of ρ to be estimated (model 7, SEIR model only).

***Hypothesis 8*** *on death from EBLV-1 infection:* infectious bats were assumed to die at the rate of *χ* to be estimated (model 8, SEIR model only).

#### (iii) Parameter estimation and model evaluation

A Metropolis-Hastings (MH) Markov chain Monte Carlo (MCMC) algorithm was implemented to estimate unknown parameters(i.e. *β*, *β*_0_, *β*_1_, *ε*, *α*, *σ*, *σ*_0_, *σ*_1_, ω, *ρ*, *χ*, ϕ and π) (electronic supplementary material, table S1). Uniform distributions (i.e. prior distributions) were used for those parameters (electronic supplementary material, table S1). Other parameter values were assumed based on field observations or previous bat-RABV studies (electronic supplementary material, table S1) [16,39,47,48].

In each MCMC iteration, parameter values were proposed from the lognormal distributions (i.e. proposal distributions). Based on those values, the proportion of seropositive bats was predicted for each colony, age group and sampling date by solving a compartmental model on a daily time scale using the *odin* function of the *odin* package [54] in R. 4.1.2 [45]. The parameter values were then accepted or not by the MH MCMC algorithm based on the following likelihood function, *L*:

2.14 $$L=\prod \mathrm{binomial}(x_{\mathrm{colony},\mathrm{age},\mathrm{date}}:n_{\mathrm{colony},\mathrm{age},\mathrm{date}},p_{\mathrm{colony},\mathrm{age},\mathrm{date}}),$$

where $x_{\mathrm{colony},\mathrm{age},\mathrm{date}}$ is the number of seropositive bats among $n_{\mathrm{colony},\mathrm{age},\mathrm{date}}$ bats sampled, stratified by colony, age and sampling date. $p_{\mathrm{colony},\mathrm{age},\mathrm{date}}$ is the proportion of seropositive bats in each category predicted by solving a compartmental model with proposed parameter values.

We iterated the models until convergence was considered achieved based on a visual inspection of MCMC trace plots and Gelman-Rubin convergence diagnostic. The models were run with four chains with random starting values. The iterations before convergence were discarded. All models across the SEIR and SEID/SER model sets were compared based on the deviance information criterion (DIC). For any two models, if their DIC difference was greater than 5, the model with a lower DIC was considered to explain the data better than another. The models whose DIC were within 5 of the lowest DIC were selected as a group of final models. In addition, model weights were computed using their DIC difference from the lowest DIC to provide the information on the relative amount of model support [51].

For the final models, we estimated the posterior predictive distribution of lyssavirus seroprevalence over the study period through simulations based on the joint posterior distribution to assess the model fit. Based on the median parameter estimates, we also simulated the number of bats in each compartment to identify periods with the highest lyssavirus transmission and estimated, over the study period, the probability of having at least one infectious bat among the median number of bats tested by RT-PCR per sampling event.

In addition, we fitted the alternative versions of the model with the lowest DIC by assuming frequency-dependent transmission and assessed their DIC and posterior predictive distributions. Finally, we performed sensitivity analysis to evaluate the robustness of our results, given that population size estimates could be affected by the frequency and timing of sampling in the year. Indeed, most samples were collected between April and May before a birth pulse, and in September, when adult bats often left the colony for mating, while the actual population size could have peaked between July and August, as observed in 2017. Therefore, using the population size ratio between July and other months observed in 2017 as the reference, the maximum population size was re-estimated for other years, and its impact on model results was assessed.

## 3. Results

### (a) Descriptive analysis results

A total of 837 bats were sampled from colony 1 (68.2%) and colony 2 (31.8%) over 27 sampling events between 2015 and 2021 and tested for neutralizing antibodies against lyssaviruses. Sampling was not conducted in 2020 in both colonies owing to the COVID-19 pandemic and in 2015 and 2018 in colony 2 for logistic reasons. The annual number of sampled bats ranged from 72 (8.6%) in 2021 to 291 (34.8%) in 2017, and the number of sampled bats per sampling event ranged from 7 to 66 with a mean of 31 (s.e.: 2.71). Most bats were adults (62.1%), females (79.3%) and *M. myotis* (91.9%) (electronic supplementary material, table S2). Around one-third of bats (36.3%) were seropositive. All bats tested by RT-PCR were negative for shedding EBLV-1 and possibly other related lyssavirus species in the saliva (*n* = 356 between 2015 and 2021 and 200 in 2022).

### (b) Logistic regression and generalized additive model results

Multivariate mixed-effect regression analysis showed that lyssavirus seropositivity was associated with *species* and *physiological status* (electronic supplementary material, table S3). *Myotis blythii* was less likely to be seropositive than *M. myotis* (odds ratio (OR): 0.30, 95% CI: 0.13–0.70, *p*-value: 0.005). Reproductively active bats were more likely to be seropositive than reproductively non-active bats (OR: 3.55, 95% CI: 1.11–11.37, *p*-value: 0.033). *Age* and *sex* were not associated with lyssavirus seropositivity. Between-year variance explained 62.8% of the total variance, while between-colony variance explained only 0.4% of the total variance (electronic supplementary material, table S3).

The analysis of the GAM showed strong evidence for intra- and inter-annual trends in lyssavirus seroprevalence, with both a cubic spline term for intra-annual variability and a random effect term for inter-annual variability significantly associated with lyssavirus seroprevalence (*p*-value < 0.001) (electronic supplementary material, table S4 and figure S2). For the variables included as fixed effects, we found no evidence for the association of lyssavirus seroprevalence except for *species* (electronic supplementary material, table S4).

### (c) Mechanistic compartmental model results

The results of our full model comparison are presented in table 1 and the electronic supplementary material. Our analysis of mechanistic compartmental models showed that SEIR model 2.1 had the lowest DIC. A total of four other alternative models had similar DIC (i.e. within five units of the lowest DIC). The relative weight of each model was as follows: SEIR models 2.1 had the highest support with a weight of 59%, followed by SIED/SER model 2.1 with a weight of 16%, and SEIR models 5, 6.1 and 6.3 with a weight of 7% each, whereas the other models had relatively low supports with only 4% as the sum of their weights (table 1). We selected these five models as our final models and present together in this study.

All of our final models supported the following transmission mechanisms, as opposed to the hypotheses of non-final models (table 1):

(i) density-dependent transmission;
(ii) higher transmission rate between synchronous birthing and hibernation than between colony forming and synchronous birthing, and lowest transmission rate during hibernation;
(iii) increase in the rate of immunity loss during hibernation; and
(iv) presence of maternity immunity.

The electronic supplementary material, tables S5 and S6 show the parameter estimates of the final models. All of the final models estimated that immunity from infection waned considerably faster during hibernation compared to before hibernation. For example, SEIR model 2.1 estimated that 82.2% of bats (95% credible interval (CrI): 79.9–84.4%) would lose their immunity from infection during hibernation, while only 8.4% (95% CrI: 7.6–10.9%) would lose their immunity before hibernation. The loss of maternal immunity also showed a similar trend, but at faster rates than immunity from infection, with 41.0% (95% CrI: 10.4–69.4%) of bats expected to lose maternal immunity before hibernation, compared to 89.4% (95% CrI: 83.5–95.3%) during hibernation in SEIR model 2.1.

**Figure 1.**
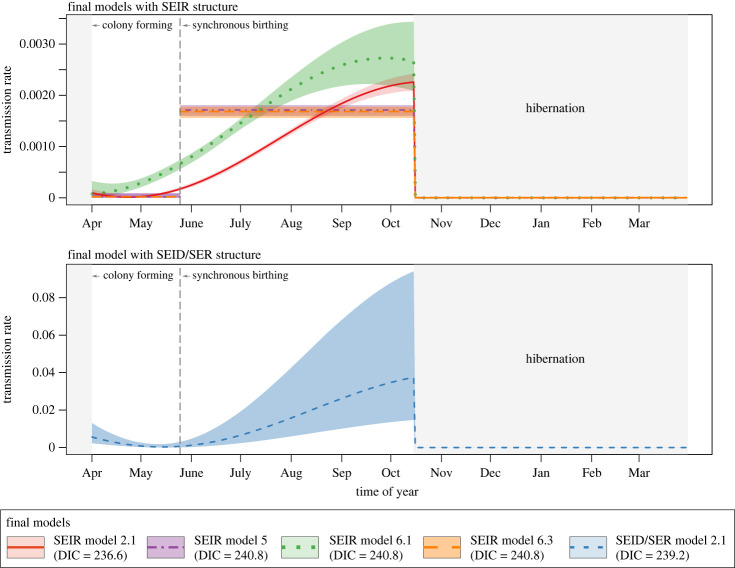
Seasonal changes in the lyssavirus transmission rate by the final models. Lines and background shades represent the median transmission rate and its 95% percentile intervals, respectively. Different colours and line types represent different final models. Vertical grey lines represent the timing of synchronous birthing, and grey shades represent hibernation periods.

The transmission rate (*β*) after synchronous birthing was estimated to be significantly higher than *β* before synchronous birthing (figure 1; electronic supplementary material, table S5). When *β* was allowed to change in a stepwise manner, *β* after synchronous birthing increased 85.8 (95% CrI: 9.6–1127.8) and 103.4 (95% CrI: 7.4–1483.9) times compared to before synchronous birthing in SEIR models 5 and 6.3, respectively. The similar trend was also observed when *β* was allowed to change continuously following a sinusoidal curve (figure 1; electronic supplementary material, table S5). In SEIR model 6.3, which estimated *β* separately for hibernation, *β* was estimated the lowest during hibernation, in line with the findings of other final models that assumed no transmission during hibernation. Finally, SEID/SER model 2.1 estimated that 5.8% of bats (95% CrI: 1.3–13.3%) exposed to the virus would become infectious and then die. As expected from the limited proportion of infectious bats, *β* was consistently estimated to be significantly larger for the final SEID/SER model than for the final SEIR models (figure 1; electronic supplementary material, table S5).

**Figure 2.**
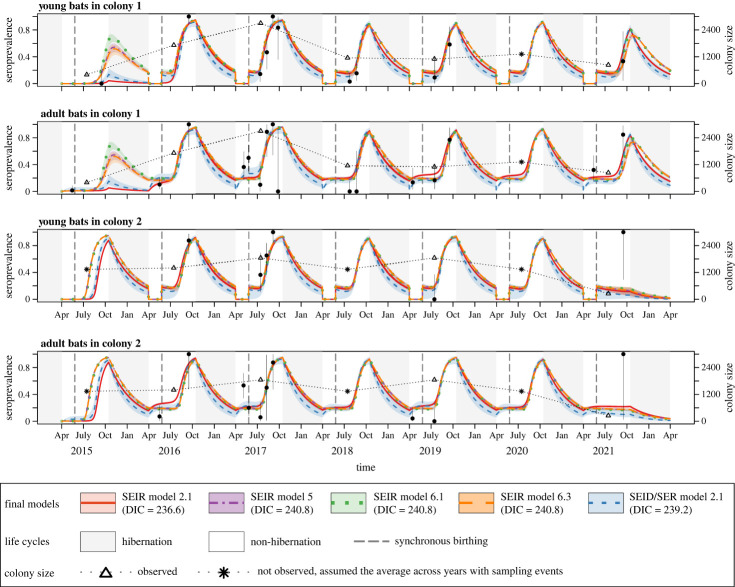
Posterior predictive distribution of lyssavirus seroprevalence for the final models. Points and vertical lines represent the observed mean and 95% confidence intervals of lyssavirus seroprevalence, respectively. Lines and shades represent the median lyssavirus seroprevalence and its 95% percentile intervals, respectively, predicted by the final models. Different colours and line types represent different final models. Grey shades correspond to hibernation periods. Triangles represent the largest population size observed each year. For years with no sampling event, the average population size across years with sampling events was assumed and shown as asterisks.

**Figure 3.**
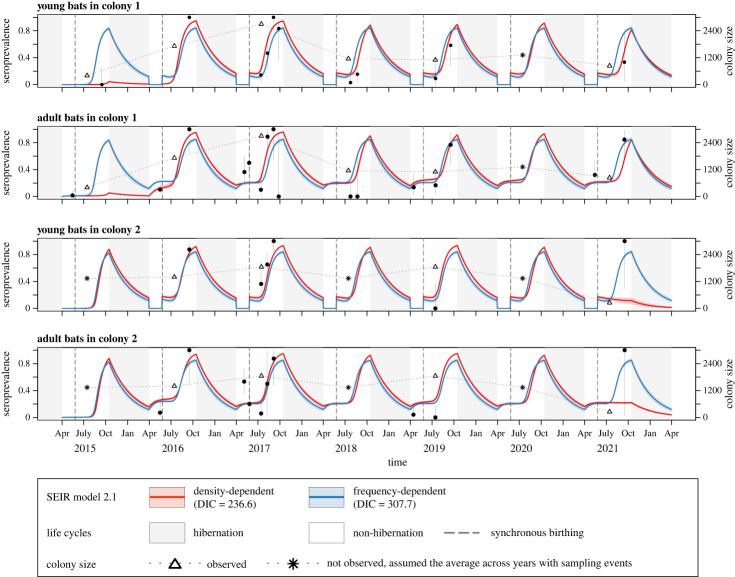
Comparison of the posterior predictive distribution of lyssavirus seroprevalence between SEIR model 2.1 assuming density-dependent transmission and the alternative model assuming frequency-dependent transmission. Points and vertical lines represent the observed mean and 95% confidence intervals of lyssavirus seroprevalence, respectively. Red lines and shades represent the median lyssavirus seroprevalence and its 95% percentile intervals, respectively, predicted by SEIR model 2.1 assuming density-dependent transmission. Blue lines and shades represent those predicted by the alternative model assuming frequency-dependent transmission. Grey rectangles correspond to hibernation periods. Triangles represent the largest population size observed each year. For years with no sampling event, the average population size across years with sampling events was assumed and shown as asterisks.

The final models predicted lyssavirus seroprevalence reasonably well, except for the 2021 trend in colony 2 for both young and adult bats (figure 2). With other assumptions kept the same, alternative models assuming frequency-dependent transmission (figure 3) or adjusting colony size based on the survey data in 2017 (electronic supplementary material, figure S3) predicted lyssavirus seroprevalence to peak every year in similar magnitudes, including in 2021 for colony 2. However, the overall predictions by these alternative models were poorer than those assuming density dependent or original colony size, as reflected by larger DIC values (figure 3; electronic supplementary material, figure S3 for DIC values).

Finally, the final models predicted a sharp peak in the number of exposed bats and the number of infectious bats between July and August each year, except for 2015 in colony 1 and 2021 in colony 2 when no outbreak was predicted to have occurred (electronic supplementary material, figure S4). These peaks were predicted to have occurred earlier with larger population sizes (electronic supplementary material, figure S5). In the final SEIR models, the median percentage of infectious bats was predicted to peak at 7.5–10.3%, contrasting to 0.5% predicted by the final SEID/SER model. Reflecting these trends, for the models assuming no heterogeneity in transmission (SEIR models 2.1, 5, 6.1 and 6.3), the probability of not capturing any infectious bats among 27 bats (i.e. the median number of bats tested by RT-PCR per sampling event) was estimated to be greater than 50% for 79.1–86.4% of the studied period, and for the model assuming heterogeneity in transmission (SEIR/SER model 2.1), all of the studied period (electronic supplementary material, figure S6). Across sampling events, the probability of capturing no infectious bats out of 27 bats ranged 7.4–100% with a median of 86.3% for the final SEIR models and increased to 87.1–99.4% with a median of 95.9% for the final SEID/SER model (electronic supplementary material, figure S6). However, with 200 bats sampled on 10 August (i.e. the number of bats sampled and tested by RT-PCR in 2022 and sampling date), the probability of capturing no infectious bats dropped to almost 0.0% by the final SEIR models and 37.1% for the final SEID/SER model (electronic supplementary material, figure S7).

## 4. Discussion

Our study aimed to understand lyssavirus transmission dynamics in mouse-eared bats by analysing longitudinal surveillance data collected from two maternity colonies in northern Italian churches over eight years. Our models, fitted to longitudinal seroprevalence data, systematically assessed lyssavirus transmission dynamics in those bats by explicitly describing the processes underlying lyssavirus transmission based on a set of hypotheses related to the disease epidemiology.

Despite the widespread distribution of lyssaviruses in European bats, only a few studies have investigated their transmission dynamics [37–39]. While providing theoretical insights into viral persistence in bats, two of these studies were mostly simulation-based and did not provide information on transmission trends within years as they analysed only annual data [38,39]. A more recent study fitted mechanistic models directly to seroprevalence data, focusing on factors required for viral maintenance in bats [37]. However, their models were still fitted to annual data. In this regard, our study extends our knowledge of lyssavirus transmission dynamics by fitting models to seroprevalence data with a sufficient temporal resolution to investigate transmission trends within and over the years. This also proved critical for understanding lyssavirus transmission dynamics in relation to bats’ life cycle events, particularly synchronous birthing and hibernation. Furthermore, the seroprevalence data linked to age-structured models allowed exploration of the consequences of infection-induced and maternal immunity loss for lyssavirus transmission dynamics, particularly in the context of having hibernation. Finally, fitting models with the SEIR or SEID/SER structure to seroprevalence data provided an opportunity to explore potential heterogeneities in lyssavirus infection in bats, identify gaps and suggest directions for future research.

Our modelling findings suggest that lyssavirus transmission is density dependent, driven by contact likely to increase with population size. Although the precise mechanisms of lyssavirus transmission between bats are still poorly understood, direct contact through biting and scratching has been hypothesized to play a major role [20]. For example, previous studies have demonstrated the infection of bats after subcutaneous or intradermal inoculation, detected lyssaviruses in bats' saliva after inoculation and observed aggressive behaviours from infected bats towards those not infected [50,55–57]. In the studied colonies, myotis bats were found tightly packed in the two colonies, despite having enough space for roosting. Such a roosting behaviour would accelerate physical contact between bats with increasing population size, thereby promoting density-dependent transmission, which is in line with our modelling findings.

We found no modelling evidence to support either homogeneous or heterogeneous transmission based on available serological data. Although SEIR model 2.1 had the highest model support, its DIC was only slightly lower than the DIC of SEID/SER model 2.1. Indeed, the fact that lyssavirus was not detected in any of the sampling events may support heterogeneous transmission, where most viral exposures do not result in viral amplification. Such an abortive infection, if present for lyssavirus infection in bats, could be associated with low-dose viral exposure and/or heterogeneous immune responses [20], as often observed in animal and human diseases [56,58,59]. Additional research is needed to validate the presence of heterogeneous lyssavirus infection and identify the factors that contribute to it.

A synchronous birth pulse has been suggested to force seasonal pathogen transmission in wild animal populations, including bats [11,60–62]. Our final models support this, as the transmission rate was estimated to become considerably higher after synchronous birthing until hibernation. This increase in the transmission rate may reflect an increase in the frequency and intensity of viral exposure (e.g. physical contacts between bats) after synchronous birthing through lactating and later mating [49], although it is not known how much bats naturally infected with lyssaviruses participate in breeding activities. Furthermore, if lyssavirus transmission was density dependent, as suggested by our findings, the force of infection would have also significantly increased when the population doubled following synchronous birthing. In addition, if female adult bats became more susceptible to lyssavirus infection during pregnancy and lactation, as commonly observed in mammals [1,63,64], it could have created favourable conditions for more effective and widespread transmission. Indeed, in 2017 when sampling was conducted most comprehensively, we observed that lyssavirus seroprevalence in both colonies was moderately high among adult bats when they first formed the maternity colonies. Then, it decreased during late pregnancy and peaked later after weaning of pups.

Our modelling analysis suggests that immunity loss is essential to seasonal lyssavirus transmission. Our findings support that both active and passive immunity wane fast enough to replenish the pool of susceptible bats each year, thereby creating favourable conditions for yearly epidemics, together with synchronous birthing. Furthermore, the models estimating a stepwise increase in the rate of immunity loss during hibernation explained the data significantly better than the models with other assumptions on the rate of immunity loss, suggesting that bats may experience dramatic lyssavirus immunity waning during hibernation. This trend is supported by the literature, where hibernation has been frequently associated with drastic innate and adaptive immune system suppressions in mammals through reduced metabolic activity and lowered body temperature [52,53].

Our modelling findings also support the presence of maternal immunity against lyssavirus in *M. myotis*. However, maternal immunity did not appear to provide sufficient herd immunity to prevent widespread transmission in young bats, as only a relatively small proportion of adult bats were estimated to be immune after hibernation. Furthermore, maternal immunity was estimated to disappear in a relatively large proportion of young bats before their first hibernation. This finding is in line with Shankar *et al*. [50], who observed RABV antibody titres rapidly diminishing in juvenile big brown bats while remaining detectable in adult bats in captivity.

Our findings related to the rate of immunity loss provide testable hypotheses for further investigations. For example, immunity waning could be tested by longitudinally measuring antibody titres in the same bats over different life cycles. In addition, the protective effect of detected antibody titres could be assessed by inoculating captive bats with the virus. However, it must be noted that there are several important constraints to those methodological approaches, particularly related to bats’ conservation status, biosafety concerns, and the feasibility of creating experimentally similar conditions for natural life cycles such as hibernation. Furthermore, as of writing, the virus responsible for the observed lyssavirus seroprevalence has not yet been characterized, as discussed below.

Our findings also suggest an association between host species and lyssavirus transmission. The higher risk of lyssavirus seropositivity in *M. myotis* compared to *M. blythii* indicates that the two species may have different susceptibilities to the virus. Alternatively, although the two species dwelt in the same places, their contact could have been segregated to some extent. As a result, following density-dependent transmission, the much smaller population size of *M. blythii* could have limited lyssavirus transmission among them compared with *M. myotis*. While mixed-effect logistic regression analyses suggest a higher risk of lyssavirus seropositivity among reproductively active bats, it was not the case after accounting for seasonal variability in lyssavirus seroprevalence in the GAM. This suggests that the association detected in the mixed-effect logistic regression analyses may reflect seasonal changes in the transmission rate among reproductively active bats, for example, through breeding activities.

Our study has some limitations. First, as discussed above, the absence of lyssavirus detection across all of the sampling events may reflect heterogeneities in lyssavirus transmission. However, although we explored the fit of SEID/SER models, the serological data alone did not provide conclusive modelling evidence for the presence of heterogeneous lyssavirus infection in bats and its role in lyssavirus transmission dynamics. To strengthen SEID/SER model analyses additional field data, such as the number of deaths and the proportion of deaths caused by lyssavirus infection, may also be required with a sufficient temporal resolution. However, obtaining such information would be challenging as it would require frequent collection and testing of dead bats while the virus is detectable. In fact, in the two colonies, the high temperature and low humidity under the wooden church roof caused bats to be found mummified soon after their death, reducing the likelihood of detecting the virus with RT-PCR. Hence, sampling must be carefully designed with background knowledge of the target colonies and sufficient field and laboratory resources to obtain information on mortality associated with lyssavirus infection.

Second, our modelling approaches could be further strengthened by collecting more thorough information on bat population dynamics. Although our study provided a unique opportunity to investigate lyssavirus transmission dynamics with a sufficient temporal resolution based on serological data, information on population dynamics was only available at the time of sampling events. While our model explained the data significantly better when assuming the original than alternative population sizes, sensitivity analysis showed that some of our model estimates were sensitive to population size. Furthermore, information on potential contact with bats from other colonies, particularly for mating and breeding, would allow for a more robust analysis of lyssavirus transmission dynamics to understand mechanisms for viral persistence and epidemics.

Finally, it is crucial to consider that negative virological findings did not allow us to characterize the virus responsible for our serological data. Furthermore, no lyssavirus has ever been detected in *M. myotis* and *M. blythii* populations in other colonies in Italy. The sera strongly neutralized EBLV-1 in our study, and our previous investigations found no neutralization of EBLV-2 in sera collected in 2015 from the same colonies [32]. Taken altogether, the possibility of cross-reactivity with yet unknown lyssaviruses cannot be excluded, particularly considering that *Myotis* bats have been frequently associated with other lyssaviruses [20]. Therefore, continued efforts are needed to characterize the virus.

In conclusion, our study provides valuable insights into the transmission dynamics of lyssavirus among mouse-eared bats in Italy. Our findings suggest that lyssavirus transmission is density dependent and is primarily driven by an increase in the transmission rate after synchronous birthing during non-hibernation periods. Our results also suggest that the loss of immunity, especially during hibernation, creates favourable conditions for annual epidemics in the bat populations studied. Our modelling framework allows us to predict the yearly peak of lyssavirus transmission, which would pose increased risk of zoonotic spillover. This information could inform the design of effective surveillance systems and risk mitigation strategies, which are particularly important for bat colonies near human residential areas, like those in the present study, where human-bat contact is frequent through various activities.

## Data Availability

The data are provided in the electronic supplementary material [65].
